# Electrochemical and computational investigation of *Cicer arietinum* extract as renewable and environmentally green corrosion inhibitor for aluminium in acidic environment

**DOI:** 10.1038/s41598-025-96141-0

**Published:** 2025-07-10

**Authors:** Hala. M. Hassan

**Affiliations:** Department of Physical Sciences, Chemistry Division, College of Science, Jazan University, P.O. Box. 114, Jazan, 45142 Kingdom of Saudi Arabia

**Keywords:** *Cicer arietinum* extract, Corrosion inhibition, Aluminium, Langmuir isotherm, Activation parameters, Monte Carlo simulation, Chemistry, Electrochemistry, Corrosion

## Abstract

This study investigates the potential of *Cicer arietinum* extract (CAE) as a cost-effective and environmentally friendly solution to prevent aluminum (Al) corrosion in acidic environments. This study aimed to identify a cost-effective and environmentally friendly corrosion inhibitor for aluminum. The effectiveness of CAE as a novel and environmentally safe inhibiting aluminum corrosion in a 1 M hydrochloric acid solution was evaluated using weight loss measurements (WL), potentiodynamic polarization (PDP), and electrochemical impedance spectroscopy (EIS). The surface morphology of Al that was corroded in the test solution was further investigated using atomic force microscopy (AFM) and scanning electron microscopy (SEM). Analysis of inhibition efficiency and kinetic data revealed the adsorption mechanism and isotherm type. The advantages of CAE include its non-toxicity, environmental friendliness, ease of preparation, and contain (O, P, and π-Bonds). The experiments showed that adding 150 ppm of CAE at 298 K resulted in an inhibition efficiency of approximately 91.1%. While increasing inhibitor concentration improved protection efficiency, higher temperatures reduced its effectiveness. The calculated low negative values of Gibbs free energy of adsorption (Δ*G*_ad_) (− 23.2, − 22.5, − 21.9, and − 21.7 kJ mol^−1^) from the Langmuir isotherm suggest that CAE adsorbs onto the aluminum surface through a physisorption mechanism. Further electrochemical studies, such as polarization techniques, are recommended to further elucidate the corrosion inhibition mechanism. The polarization curves suggest that CAE acts as a mixed-type inhibitor. Applying CAE as a protective inhibitor to metal surfaces can significantly reduce the risk of corrosion in industrial practices. Furthermore, the Density Functional Theory (DFT) method determined the quantum chemical parameters. The inhibition mechanism of Al in the aggressive solution was elucidated through a combination of experimental data, DFT calculation, and surface analysis.

## Introduction

Aluminum, a widely used metal due to its lightweight and excellent corrosion resistance under normal conditions, can still succumb to corrosion in aggressive environments such as hydrochloric acid solutions.

Constituting the third most plentiful element in the crust of the Earth, Al is a resource that may be easily used in various industrial applications, spanning from aerospace to automotive, construction, and packaging sectors^1^. Aluminum’s resistance to corrosion in multiple conditions depends on developing a dense, sticky passive oxide coating. This film, however, is amphoteric and can disintegrate significantly in either alkaline (pH > 9) or acidic (pH < 5) conditions. Aluminium is strongly corroded by HCl, which is typically used for industrial acid cleaning, chemical or electrochemical etching, and acid pickling. To prevent aluminium from corroding in HCl, inhibitors must be found. Various organic compounds, including aliphatic amines, which include N, S, and O atoms, are effective corrosion inhibitors for aluminium in HCl conditions up to this point^2^.

The corrosion behavior of pure aluminum and its alloys in aqueous alkaline solutions has been extensively studied in developing the aluminum anode for the aluminum/air battery^3^. The corrosion of aluminium in batteries leads to numerous issues: The dissolved Al^3+^ ions migrate to the counter anode and deposit reductively, the cathode active material is passivated, its solid products increase electrical resistance, and its soluble products contaminate the electrolyte and accelerate self-discharge^4^. Despite their innate resistance to corrosion, facilitated by forming a protecting oxide layer, aluminium materials can undergo degradation over time, resulting in functional impairment and structural deterioration^5–8^.

Plant extracts as corrosion inhibitors have recently gained popularity because of their potential effectiveness, affordability, and environmental friendliness. Plant extracts encompass a variation of organic composites such as flavonoids, polyphenols, alkaloids, and tannins, which have confirmed corrosion protection properties^9–15^.

The ability of natural compounds derived from plants to prevent corrosion has drawn attention in recent years due to growing environmental consciousness and the necessity to create eco-friendly procedures. This field of study is significant since plant extracts are not only cheap, easily accessible, and renewable sources of materials, but they are also environmentally acceptable and friendly. Amongst others, extracts of some plants such as gongronema latifolium extract^16^, kola nitida extract^17^, and piper guineense extract^18^, akuamma seed extract^19^. The ability of extracts to protect against corrosion on Al alloys in a variety of conditions have been the subject of numerous investigations. In sustainable material protection goods, phytocompounds and extracts derived from herbs have attracted growing attention. The total phenolic content should be the main focus of evaluating certain plant extracts and the relationship between the extract profile and the corrosion-inhibiting activity. A strong association was found between the plant extracts’ total phenolic content and how well they inhibited corrosion. The extracts’ ability to inhibit positively correlates with their total phenolic content. One could argue that the overall phenolic content could direct the process of evaluating plant extracts for their inhibitory effects^20–23^.

Many studies have investigated the potential of these extracts, particularly on aluminum and its alloys, across various corrosive media. There’s growing interest in using plant-derived compounds for sustainable material protection. A key focus in evaluating these extracts is their total phenolic content, as a strong correlation exists between this content and corrosion inhibition effectiveness. Higher phenolic content generally leads to better inhibition. Therefore, total phenolic content can be a helpful indicator when screening plant extracts for their corrosion-inhibiting properties.

Plant extracts act as corrosion inhibitors by covering the metal surface with a protective layer that prevents corrosion. The specific processes that underlie inhibition might include phytochemicals adhering to the metal surface, preventing corrosive substances from entering and stabilising the passive oxide layer^24–28^.

CAE is a crucial legume crop with abundant protein, carbohydrates, fats, fibre, isoflavones, and minerals. CAE is highly nutritional and is utilized as a high-energy and protein source in human diets^29^.

This study offers further details on the potential use of *Cicer arietinum* extract (CAE) as an eco-friendly corrosion inhibitor under the specified conditions. The present work aims to assess the %IE of CAE for aluminium in 1.0 M HCl acid utilizing WL, PDP, and EIS techniques. Furthermore, the morphology of the aluminium surface post-immersion in 1.0 M HCl solution attendance and nonattendance CAE will be evaluated using SEM and AFM. Finally, the inhibition mechanism of CAE for aluminium will be discussed based on the experimental data, showcasing its environmentally friendly inhibitor could find possible applications in metal surface protection.

## Experimental materials and techniques

### *Cicer arietinum* extract (CAE) extract Preparation

**Fig. 1:**
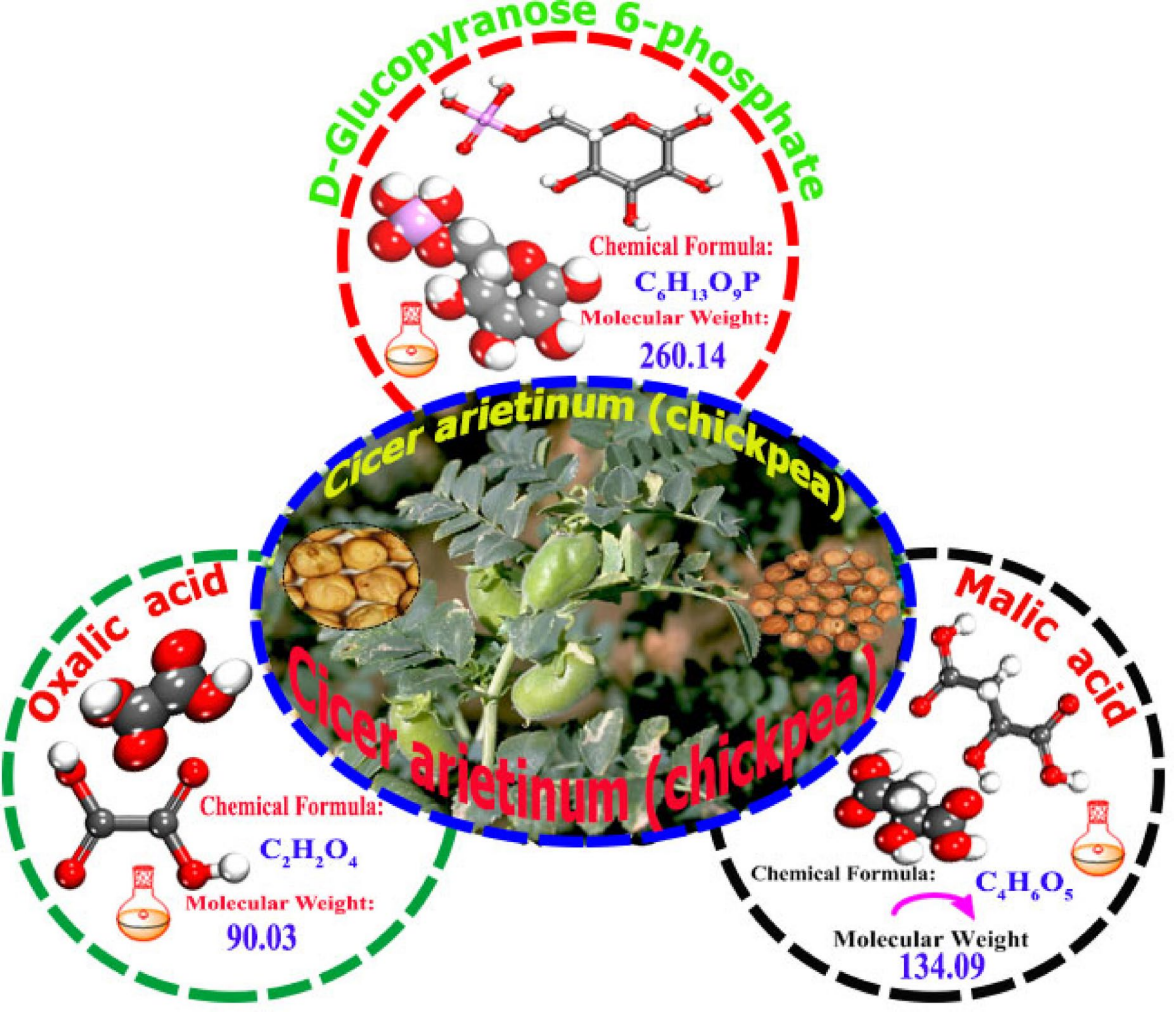
A drawing of a *Cicer arietinum* plant and its chemical components (Microsoft 365PowerPoint, https://www.microsoft.com/en-us/microsoft-365/powerpoint.

The CAE plant constituents were collected manually and washed with distilled water to remove dust or other detached residues. The CAE plant components were ground into a fine powder using an electronic mill shaded at room temperature. Then, 20 g of the powder was put in a 500-millilitre measuring flask dissolved in bidistilled water. After the 12-h soaking period, the chickpeas were thoroughly rinsed with water. The lab-scale food processor was used to grind the soaked chickpeas for one and a half minutes using varying water to chickpea ratios. “According to the experimental design, the chickpea-water slurry was heat-treated at various time and temperature combinations after grinding. The slurry was finally filtered through double-layer cheesecloth, and the collected filtrate was homogenized for stability using a homogenizer (Fisherbrand 850). The obtained filtrate was cooled and stored at 4°C for further analysis”. After evaporation, a solid extract was produced, which was thereafter ready for application as a corrosion inhibitor^30^. As shown in Fig. 1, numerous analyses have revealed that the primary chemical ingredients derived from *Cicer arietinum* extract are made up of three major organic acids: glucose-6-phosphate (5.5%), oxalate (28.6%), and malate (61.2%)^31^.

### Materials and solution Preparation

All specimens used in this examination were taken from aluminum, and a purity of 99.99% was used. To remove any remaining contaminants from the surface, the Al samples were polished using sandpaper of different grit levels and then rinsed with deionized water and alcohol. The corrosive media was 1.0 M HCl solution made from 37% analytical grade HCl. The concentration range of CAE extract employed in this investigation comprised 25, 50, 75, 100, 125, and 150 ppm, respectively.

### Corrosion tests

#### Weight loss (Wl) measurements

WL method was used to assess CAE efficacy as a corrosion hindrance for (Al) in a corrosive solution (1.0 M HCl). Mass loss studies used an Al sample calculating 2 × 2 × 0.1 cm. Initially, emery sheets of grades 300, 600, and 1500 were used to abrade the Al samples. A digital balance was then used to weigh them after they had been degreased with acetone and left to dry. After that, the samples were submerged in the test solution for varying amounts of time (30, 60, 90, 120, 150, and 180 min) at temperatures between 298 and 313 K, with or without varying concentrations of the CAE inhibitor. The samples were removed from an acidic corrosive environment after immersion, cleaned, and dried before being weighed again. During the experiment, a thermostat water bath kept the temperature at the appropriate degree. The corrosion rate (CR, mg cm^−2^ min^−1^) was calculated according to the following equation^32^:

1 $$CR=\frac{\varDelta W}{At}$$

(∆W = W1 − W2) shows the average weight loss of Al following the time (t), and A denotes the area of the Al (cm^2^).

#### Electrochemical tests

A platinum sheet (1.0 cm^2^) was used as the counter electrode, a saturated calomel (SCE) was used as the reference electrode, and an aluminum (Al) working electrode was used in the electrochemical studies. The corrosion potential was assessed using PDP experiments, which were performed at a scanning rate of 1.0 mV/s and ranged from − 1300 mV to − 100 mV. The open circuit potential (OCP) was attained after the Al working electrode was submerged in the corrosive solution for 30 min at 25 °C.

At the OCP, EIS measurements were conducted with an AC amplitude of ± 10 mV throughout a frequency range of 100 kHz to 10 Hz. A Gamry Instrument Potentiostat/Galvanostat/ZRA instrument was used for these electrochemical studies.

The inhibition efficiency (${\%IE}_{WL}$, $\:{\%IE}_{PP},\:\text{and}\:{\%IE}_{EIS}$) and surface coverage (θ) were determined from WL, EIS analyses, and potentiodynamic polarization (PDP), respectively, using the following equations^33–36^:

2 $${\%IE}_{WL}= \theta \times 100=\left[1-\frac{{CR}_{inh}}{{CR}_{free}}\right] \times 100$$

3 $${\%IE}_{PDP}=\theta \times 100=\left[1-\frac{{\text{i}}_{\text{c}\text{o}\text{r}\text{r}\left(\text{i}\text{n}\text{h}\right)}}{{\text{i}}_{\text{c}\text{o}\text{r}\text{r}\left(\text{f}\text{r}\text{e}\text{e}\right)}}\right] \times 100$$

4 $$\%{IE}_{EIS}=\theta \times 100=\left[1-\frac{{\text{R}}_{\text{p}\left(\text{f}\text{r}\text{e}\text{e}\right)}}{{\text{R}}_{\text{p}\left(\text{i}\text{n}\text{h}\right)}}\right] \times 100$$

Where CR_free_ and CR_inh_ are the corrosion rates without and with the CAE; ${\text{i}}_{\text{c}\text{o}\text{r}\text{r}\left(\text{f}\text{r}\text{e}\text{e}\right)}$ and ${\text{i}}_{\text{c}\text{o}\text{r}\text{r}\left(\text{i}\text{n}\text{h}\right)}$ represents the current without/with CAE, respectively. ${\text{R}}_{\text{p}\left(\text{i}\text{n}\text{h}\right)}$ and ${\text{R}}_{\text{p}\left(\text{f}\text{r}\text{e}\text{e}\right)}$ symbolize the charge transfer resistances with/without CAE, respectively^37^.

### Surface morphology of metal^38^

#### Scanning electron microscope measurements

The morphology of the Al surfaces was examined using a JEOL Neoscope JCM-5000 microscope after immersion in hydrochloric acid (one molar) solution for 24 h without and with the optimum concentration of inhibitor CAE.

#### Atomic force microscope (AFM)

The surface profile roughness of the (Al) samples immersed in 1.0 M HCl, both inhibited and uninhibited CAE present was investigated using the AFM technique. The samples were taken out of the test solution after a 24-h immersion period, cleaned, rinsed with bi-distilled water, and dried. A Pico SPM2100 AFM instrument was then used to analyse the aluminium samples.

### Theoretical calculation

#### Density functional theory (DFT)

The theoretical calculations are necessary to simulate the experimental data. The quantum chemical calculations were done with the Materials Studio program version 7.0 software, with a 6-31G basis set and B3LYP correlation functional. This software employs a semi-empirical approach based on Density Functional Theory (DFT) to perform simulations on various materials. Materials Studio is a powerful tool encompassing functionalities for quantum mechanics, molecular dynamics, bioinformatics, chemical informatics, and computational chemistry^39,40^.

#### Monte‑Carlo simulations (MC)

Monte Carlo simulations (MC) are known to be very important for investigations on corrosion inhibition. This simulation was performed using the Materials Studio Softwar. Researchers used the Monte Carlo (MC) method to assess how effectively CAE inhibitor positioned themselves on the Al surface. Aluminum (111) was chosen for this evaluation due to its stable crystal structure. The MC simulation optimized the arrangement of water and inhibitor molecules using an equation of motion. A compass force field was employed to simulate the interaction between the inhibitor, the water molecules, and the Al (111) surface representing the CAE. This method helped researchers identify the most favourable adsorption sites on the substrate-adsorbate system by gradually lowering the simulated temperature, effectively mapping the configuration space.

## Results and discussion

### WL test

**Fig. 2:**
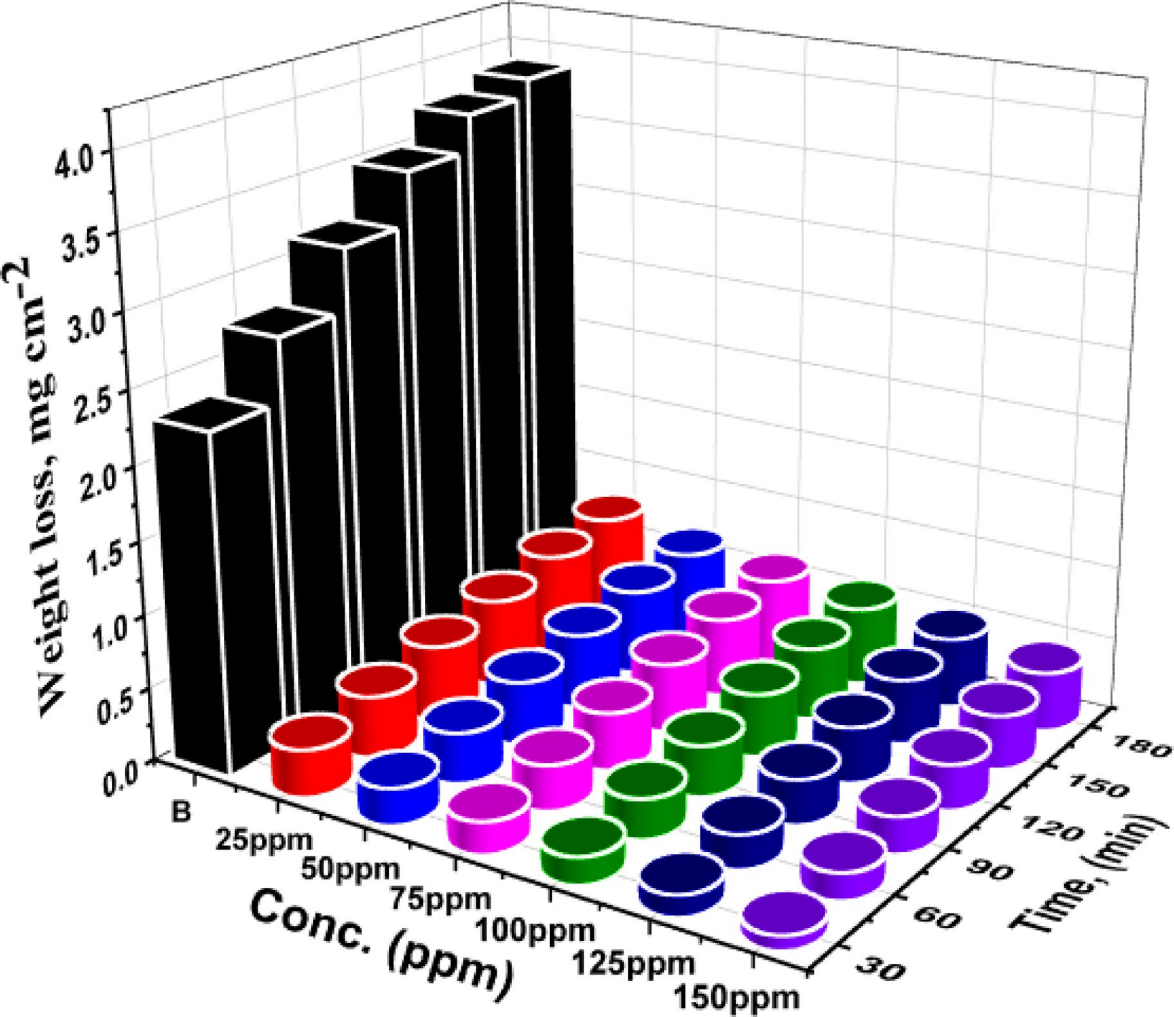
WL -time bends for Al in corrosive solution without and with CAE at 298 K.

For weight loss analysis, Al samples were produced in accordance with ASTM G 31-72^41^. WL measurements examined the corrosion inhibition effectiveness of CAE against aluminum (Al) corrosion in 1 molar HCl at various temperatures. A direct relationship between the percentage of IE and the concentration of CAE extract was found when the corrosion parameters were computed and presented in Table 1. The WL of Al samples in 1.0 M HCl over time, with and without varying doses of CAE at 298 K (Fig. 2), shows that CAE results in decreased WL compared to the free acid condition. The results show that concentration significantly influences the Al samples’ weight loss. This relationship is caused by raised inhibitor adsorption on the aluminium surface, forming a barrier layer between the aggressive solution and the Al surface. As a result, the surface coverage (θ) improves, which raises the percentage IE^42,43^.

**Table 1:** Effect of CAE doses on CR, Θ and (% IE) for al in 1.0 M HCl.

Temperature (°C)	[Inh] ppm	CR × 10^−2^ (mg cm^−2^ min^−1^)	θ	% IE
25	0.0	2.9249	–	–
	25	0.5065	0.827	82.7
	50	0.4335	0.852	85.2
	75	0.3980	0.864	86.4
	100	0.3643	0.875	87.5
	125	0.3197	0.891	89.1
	150	0.2611	0.911	91.1
30	0.0	5.3646	–	–
	25	1.1695	0.782	78.2
	50	0.9978	0.814	81.4
	75	0.8637	0.839	83.9
	100	0.7778	0.855	85.5
	125	0.6867	0.872	87.2
	150	0.5043	0.906	90.6
35	0.0	12.6042	–	–
	25	3.7434	0.703	70.3
	50	2.5838	0.795	79.5
	75	2.2436	0.822	82.2
	100	1.9788	0.843	84.3
	125	1.6512	0.869	86.9
	150	1.2983	0.897	89.7
40	0.0	24.0583	–	–
	25	7.3859	0.693	69.3
	50	5.9183	0.754	75.4
	75	4.7876	0.801	80.1
	100	3.9937	0.834	83.4
	125	3.4885	0.855	85.5
	150	3.0313	0.874	87.4

#### Temperature effect and kinetic parameters

The difference of (CR) and (%IE) of CAE extract on Al in 1 molar hydrochloric acid with the change in temperature and dose of CAE is signified in Table 1. The (CR) rises as the temperature increases, and the (%IE) falls. The reduction in the adsorption process indicates that the CAE molecules’ desorption from the Al surface has been accelerated by the increased temperature^44,45^.

This behavior confirms that the process of adsorbed CAE on the surface of Al is carried out through physisorption with weak bonds. Using Arrhenius and transition-state equations, the kinetic activation parameters for Al dissolution in one molar HCl solutions with and without different concentrations of CAE extract throughout the temperature range of 298–313 K are determined as follows^46–49^:

5 $$\text{ln }CR=\text{ln }A- \frac{{E}_{a}^{*}}{RT}$$

6 $$\text{l}\text{n}\frac{CR}{T}=\text{ln}\frac{R}{Nh}+\frac{{\varDelta S}^{*}}{RT}-\frac{{\varDelta H}^{*}}{RT}$$

The activation energy, entropy, and enthalpy are represented by E*a, ∆S*, and ∆H*, respectively, where A signify the Arrhenius pre-exponential factor, h is the Planck constant, and N mean Avogadro’s number.

**Fig. 3:**
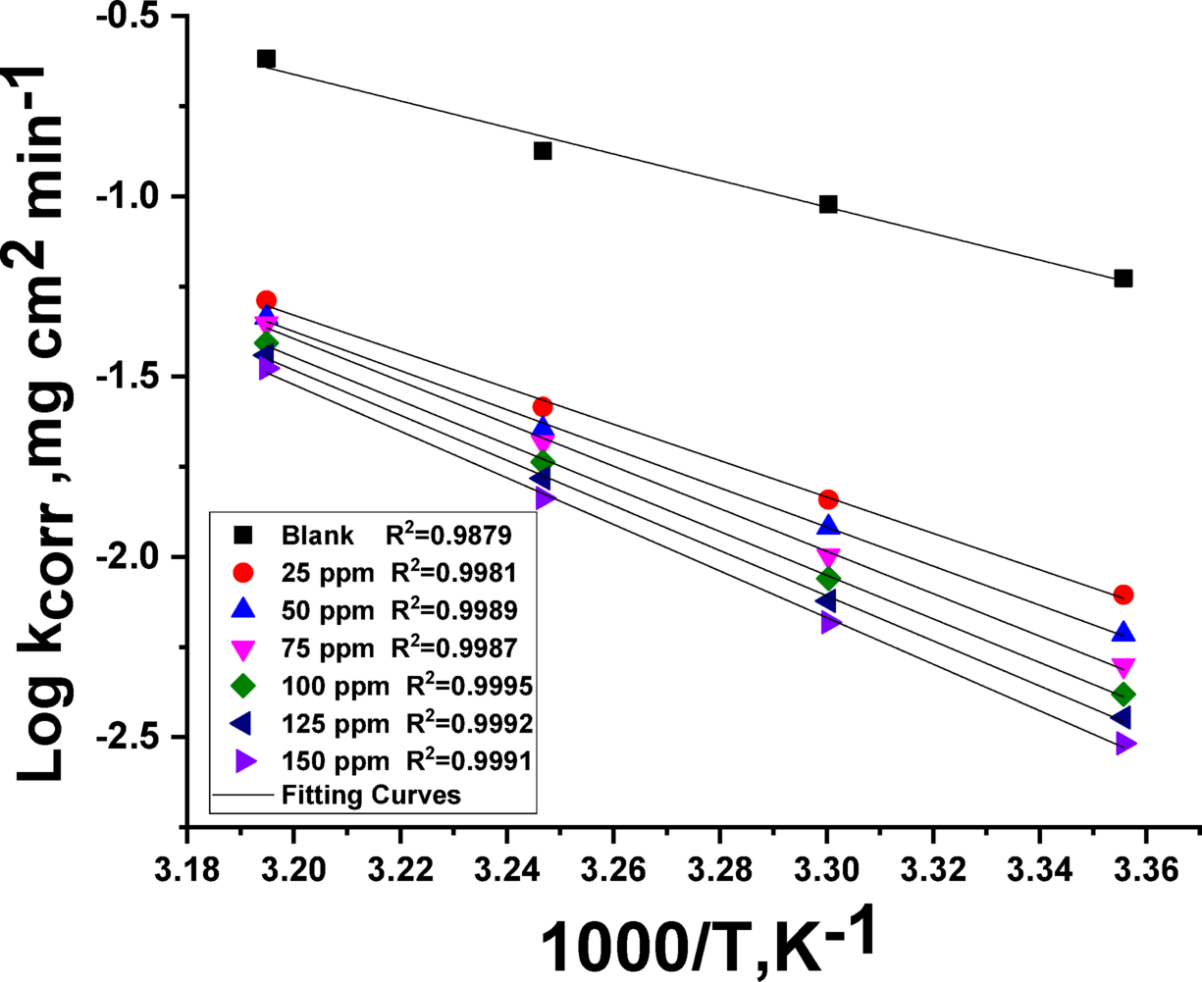
log k_corr_ vs.1000/T bends for altered doses of CAE.

**Fig. 4:**
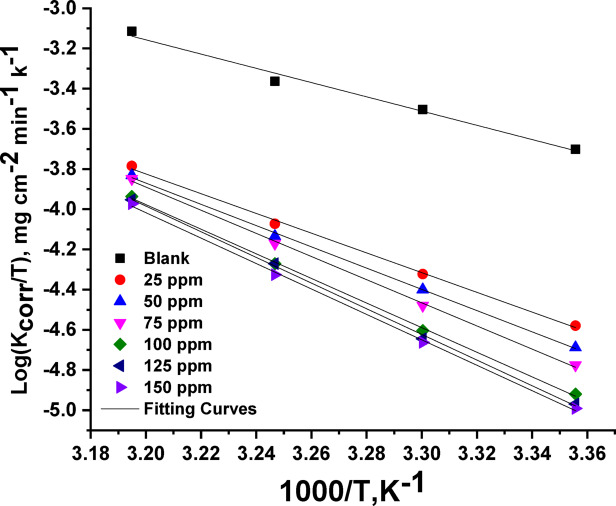
log (k_corr_/T) vs. 1/T bends for various doses of CAE.

According to the Arrhenius equation (Eq. 5), the activation energy for the Al dissolution process in 1 M HCl at varying doses of CAE was calculated using the linear fitted slope of log CR vs. 1/T (Fig. 3). Furthermore, ∆H* and ∆S* of activation were calculated using the slope and intercept of the straight lines obtained from log CR/T vs1/T, respectively, in accordance with the transition-state equation (Eq. 6) (Fig. 4).

As seen from the data in Table 2, the present investigation shows that the energy barrier of the dissolution reaction rises in the existence of CAE, leading to an increase in E*a in the presence of CAE inhibitor. Two possible explanations exist for this increase: physical adsorption or a decrease in inhibitor molecule adsorption on the Al surface as the temperature rises^50^.

**Table 2:** Kinetic parameters Al liquefaction of Al in one molar HCl with altered doses of CAE.

Conc., ppm	E_a_^*^, kJ mol^−1^	∆H^*^, kJ mol^−1^	−∆S^*^, J mol^−1^ K^−1^
Blank	70.4	67.9	40.7
25	96.5	93.9	45.4
50	103.8	101.2	52.3
75	112.7	110.2	70.8
100	115.8	113.3	88.7
125	119.7	117.2	92.3
150	123.6	121.1	98.6

Values of (E^*^_a_) behave similarly to values of ∆H*. Enthalpy values in the inhibited solution are higher than those in the reference electrolyte, indicating increased prevention efficiency and rapid dissolution of Al in an uninhibited solution. Furthermore, the endothermic character of the Al dissolution process is demonstrated by the positive values of ∆H* in the treated and untreated solutions. In the presence of CAE, Table 2 also shows negative entropy values. These negative values imply that an association process, rather than a dissociation process, is involved in the activation complex’s rate-determining steps^51–53^.

#### Adsorption isotherms and thermodynamic parameters

**Fig. 5:**
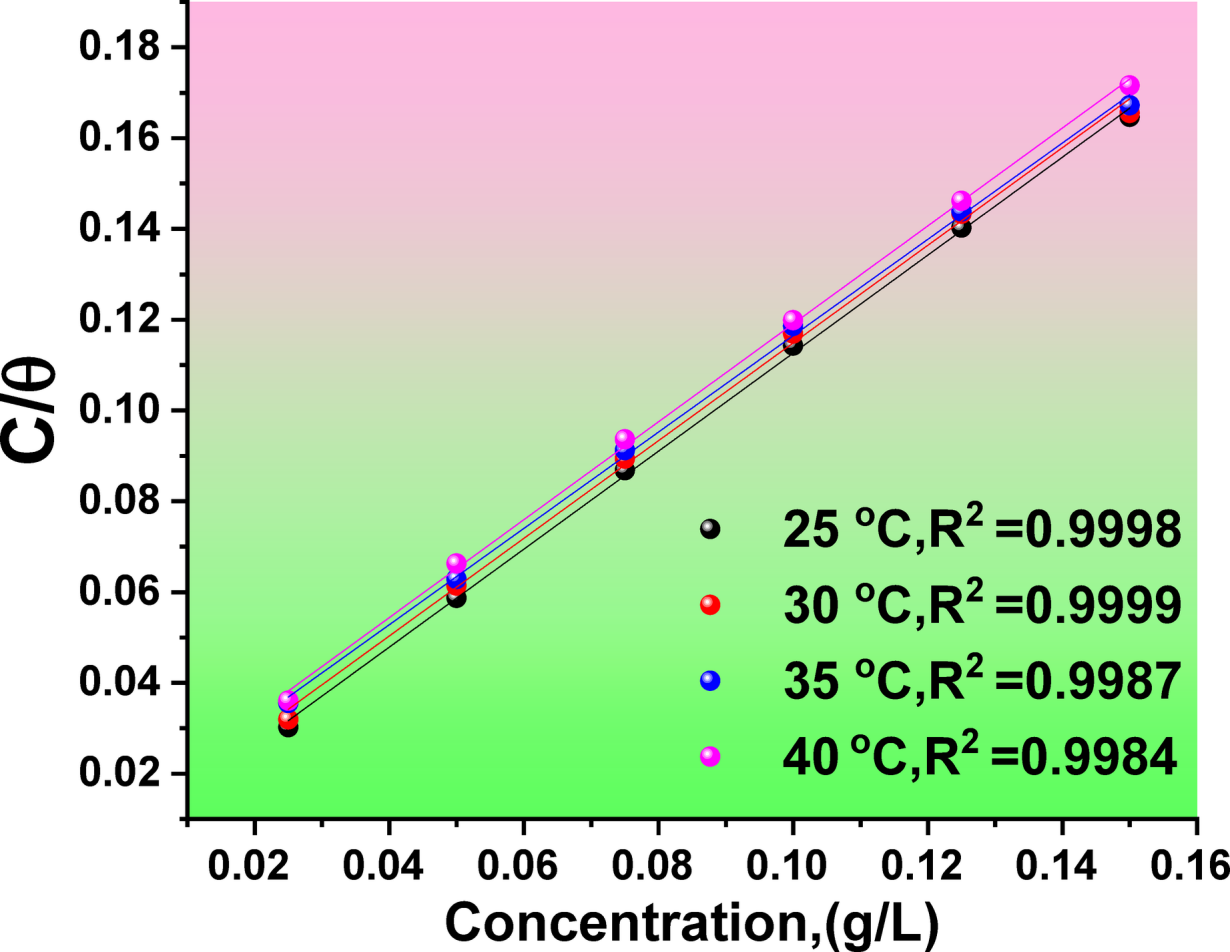
Langmuir adsorption plots at different temperatures.

Many adsorption isotherms, including the most often utilized ones, including Langmuir, Henry, Temkin, and Friendly, control the interaction between the CAE and the Al surface. The present study analysed different adsorption isotherms to find the best fit. The Langmuir isotherm was the most suitable one, producing the best linear plots, as shown by correlation coefficients (R^2^) near unity, confirming the validity of this isotherm, as shown in Fig. 5. The following equation represents all these adsorption isotherm^54,55^

7 $${\text{Langmuir}}\quad \frac{{{C_{inh}}}}{\theta }=\frac{1}{{{K_{ads}}}}+{C_{inh}}$$.

8 $${\text{Henry}}\quad \theta ={K_{ads}}C$$

9 $${\text{Temkin}}\quad \alpha \theta =\ln {K_{ads}}C$$

10 $${\text{Friendlish}}\quad \log \theta =\log {K_{ads}}+n\log C$$

The adsorption constant (K_ads_), CAE concentration (C), surface coverage (θ) for altered concentrations, and α is a factor that describes the molecular interactions in the adsorption layer and surface heterogeneity, were all determined using the weight loss method. Plotting θ derived from WL tests against CAE concentration is done in every instance. We can evaluate which adsorption isotherm model best fits the experimental data by measuring the straight lines’ correlation coefficient (R2). The adsorption of CAE onto the Al surface appears to have closely adhered to this Langmuir isotherm, based on the significant correlation (R^2^ = 0.99) found. Figure 5 displayed the CAE drawing at change temperatures as (C) vs. (C/θ). The adsorption constant results in the ∆G°_ads_ by next, with the intercept equal to (1/K_asd_) and the slope around unity.

11 $$\varDelta {G}_{ads}^{\circ} = {-RTln\:(55.5\:K}_{ads})$$

The ∆G°_ads_ data at all temperatures are documented in Table 3. The negative (∆G°_ads_) value shows the adsorption process’ spontaneity. The (∆G°_ads_) data suggest that CAE’s adsorption mechanism on metal in 1.0 M HCl is physical adsorption. The (∆H°_ads_.) was a measure approving the Van’t Hoff balance^56^.

12 $$\text{log}{k}_{ads} =\left(\frac{{-\varDelta H}_{ads}^{^\circ }}{2.303RT}\right) +constant$$

**Table 3:** Thermodynamic parameters of CAE.

Temp, K	K_ads_ M^−1^	−ΔG°_ads_ kJ mol^−1^	−ΔH°_ads_ kJ mol^− 1^	−ΔS°_ads_ J mol^−1^ K^−1^
298	211	23.2	53.2	77.7
303	136	22.5		74.1
308	96	21.9		71.2
313	76	21.7		69.3

**Fig. 6:**
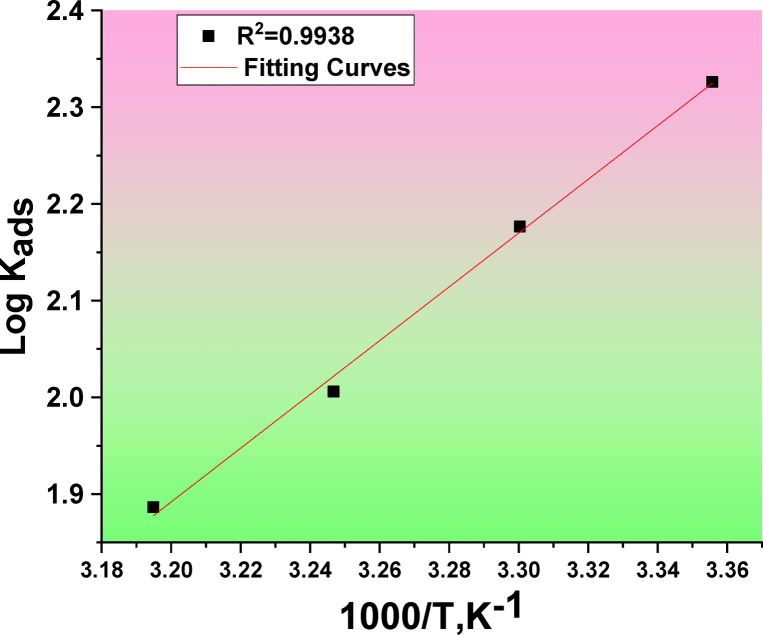
Plot (log K_ads_) vs. (1/T) for the corrosion of Al in existence of CAE at altered temperatures.

As shown in Fig. 6, a linear connection was obtained by plotting log k_ads_ versus 1/T. The enthalpy of adsorption (∆H°_ads_) was calculated and documented in Table 3 based on the slope of this line, which is (− ∆H°_ads_/2.303R). The equation that follows was then used:

13 $$\Delta {\text{G}}{^\circ _{{\text{ads}}}}={\text{ }}\Delta {\text{H}}{^\circ _{{\text{ads}}}} - {\text{ T}}\Delta {\text{S}}{^\circ _{{\text{ads}}}}$$

The values of (ΔS°_ads_) were calculated. The adsorption of CAE extract appears to be mostly physical and exothermic, based on the negative (ΔS°_ads_) value. Additionally, the –ve sign (ΔS°_ads_) is attributed to increased solvent entropy as disorder occurs at the Al/solution interface. This phenomenon is explained by the displacement of H_2_O molecules by CAE molecules on the surface of Al in the test medium^57^.

### Electrochemical measurements

#### Open circuit potential (OCP)

**Fig. 7:**
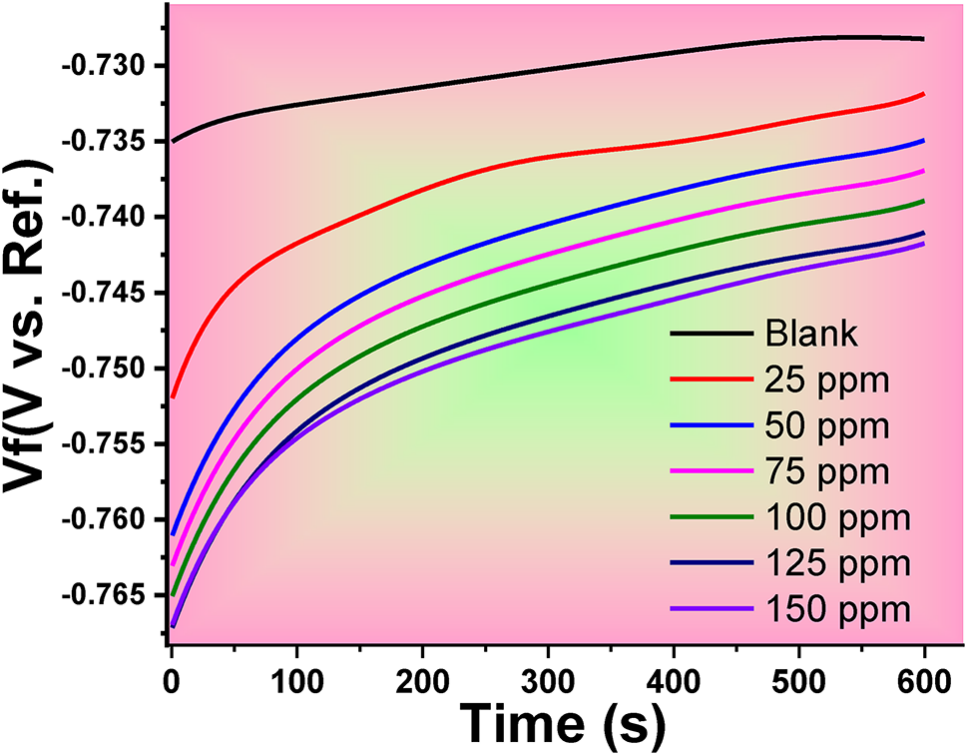
E_OCP_ versus time variation for Al in the 1.0 M HCl solution with and without different CAE concentrations.

The OCP time dependency obtained for immersion of the Al in a 1 M HCl solution, both with and without the optimum concentration of CAE, is displayed in Fig. 7. For all corrosion studies, OCP monitoring is essential since EIS and PDP tests happen at OCP by forcing the system away from equilibrium. After a hundred seconds of immersion, the OCP levels were comparatively steady. The variation and variance in OCP at the start of the exposure may be due to the aggressiveness of the hydrochloric acid solution and the creation of an oxide layer. This indicates that as the potential falls, the rate of oxide thickening also rises. In this phase, the protective coatings that shield the metals form^58,59^.

#### PDP tests

**Fig. 8:**
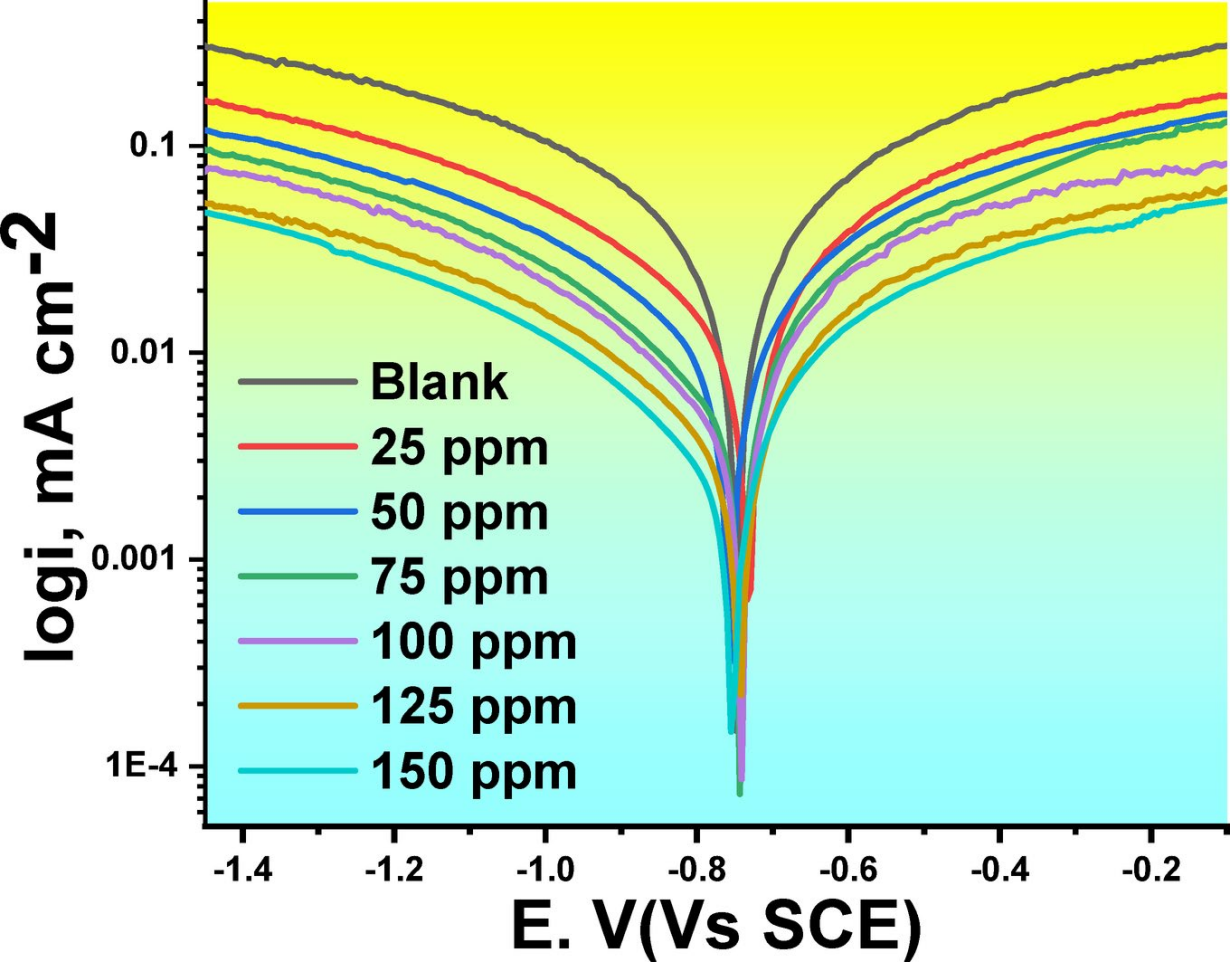
PDP curves of Al liquefaction in 1.0 HCl with/without altered doses of CAE.

Figure 8 shows the polarisation diagrams for Al in one molar HCl solution with and without different dosages of CAE extract at 298 K. CAE inhibitor decreases the current density for both anodic dissolution reaction (Al → Al^3+^ + 3e-) and cathodic hydrogen evolution reaction (2 H^+^ + 2e- → H_2_↑) when compared to the blank solution (without inhibitor). The linear segments of the anodic and cathodic branches of the Tafel slopes were extrapolated to determine the corrosion current density (i_corr_). The current density values (*i*_*corr*_) decreased with increasing concentration of CAE, from 259 mA cm^−2^ in 1 M HCl to 23 mA cm^−2^ with CAE. The results indicate that the existence of CAE reduces the anodic dissolution of Al^3+^ and slows the evolution of the H^+^ ion discharge, which can be explained by an adsorption film on the surface of metal^60,61^.

The percentage of IE, θ, E_corr_, i_corr_, and Tafel slopes (βa and βc) for CAE concentration are shown in Table 4. The corrosion current densities (i_corr_) decrease with the addition of CAE extract. The % IE rises with increasing CAE concentration. At 150 ppm, it reaches 91.1% due to CAE extract molecules adhering to the Al surface.

**Table 4:** (PDP) parameters for the corrosion of al with/without of CAE at 298 K.

_[Inh]_ _ppm_	−E_corr_ mV vs. SCE	i_corr_ mA cm^− 2^	β_a_ mVdec^− 1^	β_c_ mVdec^− 1^	k_corr_ × 10^3^ mpy	θ	% IE
0	748	259	797	984	110	–	–
25	732	54	579	764	32.7	0.791	79.1
50	757	48	740	718	22.4	0.814	81.4
75	743	41	259	713	9.9	0.841	84.1
100	742	35	243	700	8.1	0.864	86.4
125	744	28	265	657	6.2	0.891	89.1
150	753	23	267	518	4.9	0.911	91.1

Interestingly, the Tafel slopes (βa, βc) do not alter with varying doses of CAE extract, suggesting that the addition of the extract has no effect on the corrosion mechanism and that the main cause of inhibition is the adsorption of extract molecules, which block active sites on the electrode surface^62^. When the corrosion potential changes by less than ± 85 mV, it is referred to as mixed type. The about 25 mV difference between the lowest and highest E_corr_ indicates the CAE extract’s mixed type character^63–66^. Ultimately, the potentiodynamic results are consistent with the WL technique results.

#### EIS tests

**Fig. 9:**
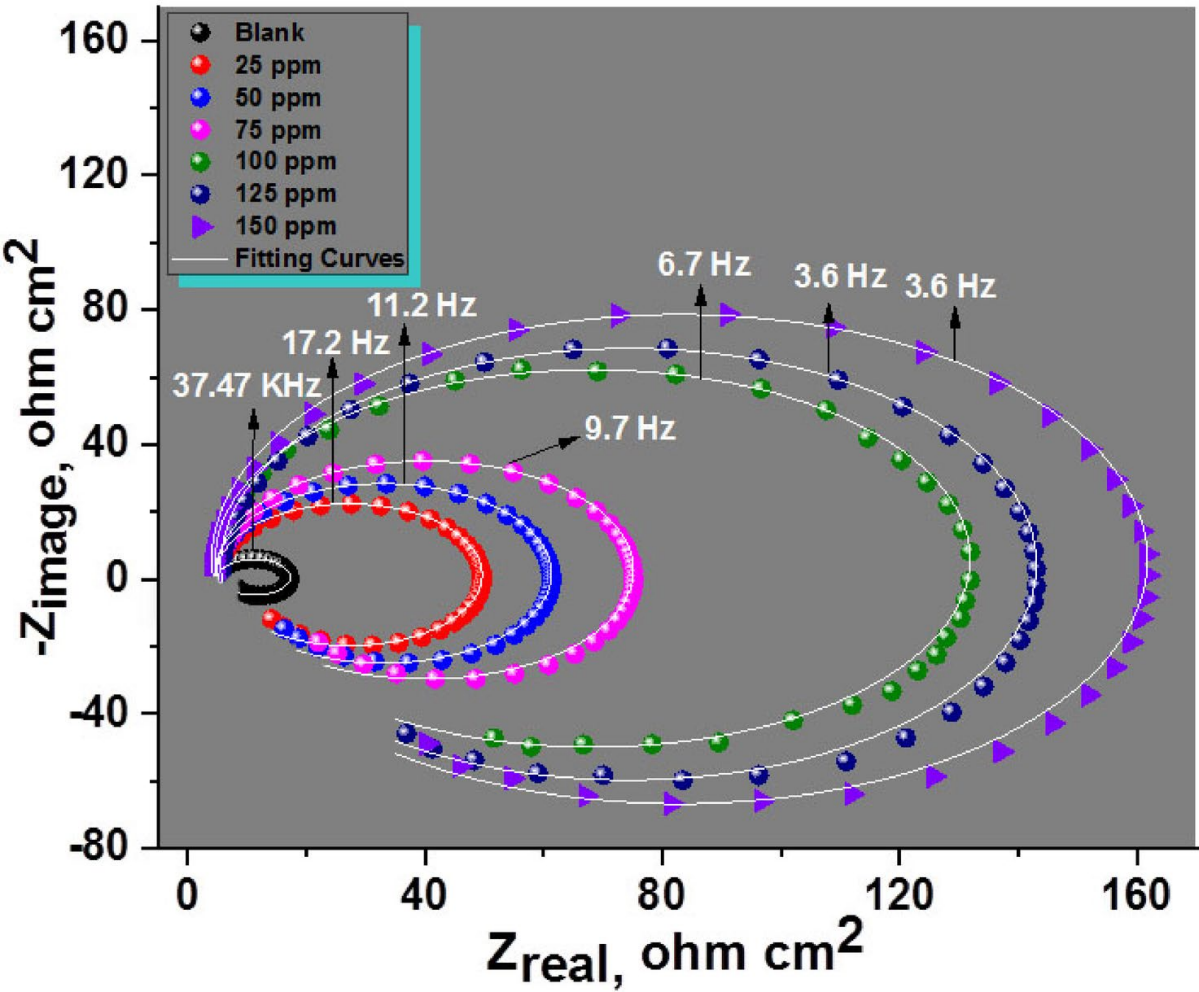
Nyquist bends for Al composite in 1 M HCl arrangements in the lack and existence of diverse doses of CAE at 25 °C.

**Fig. 10:**
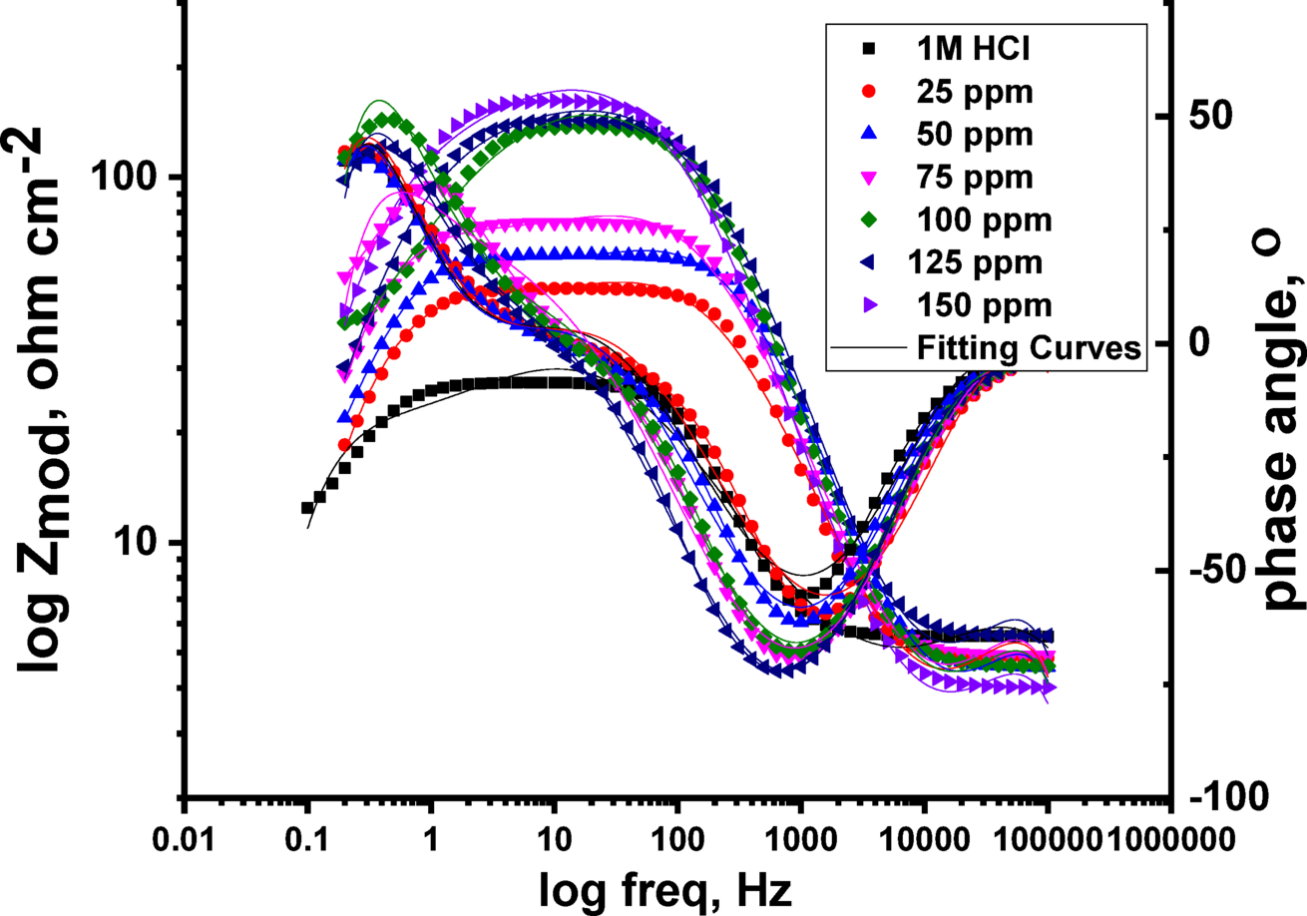
Bode curves for Al with and without diverse CAE doses at 25 °C.

Nyquist and Bode charts showing the corrosion behaviour of Al in a 1.0 M HCl solution, both attendance and lack different dosages of CAE, at 298 K, are shown in Figs. 9 and 10. The Nyquist plots show Different impedance patterns, with a smaller inductive loop at low frequencies and a larger capacitive loop at high frequencies. Because CAE adsorbs on the aluminium surface and forms a protective coating that prevents the electrode surface from dissolving in the aggressive test solution, it is significant that the semi-circular diameter rises with greater CAE concentrations.

**Fig. 11:**
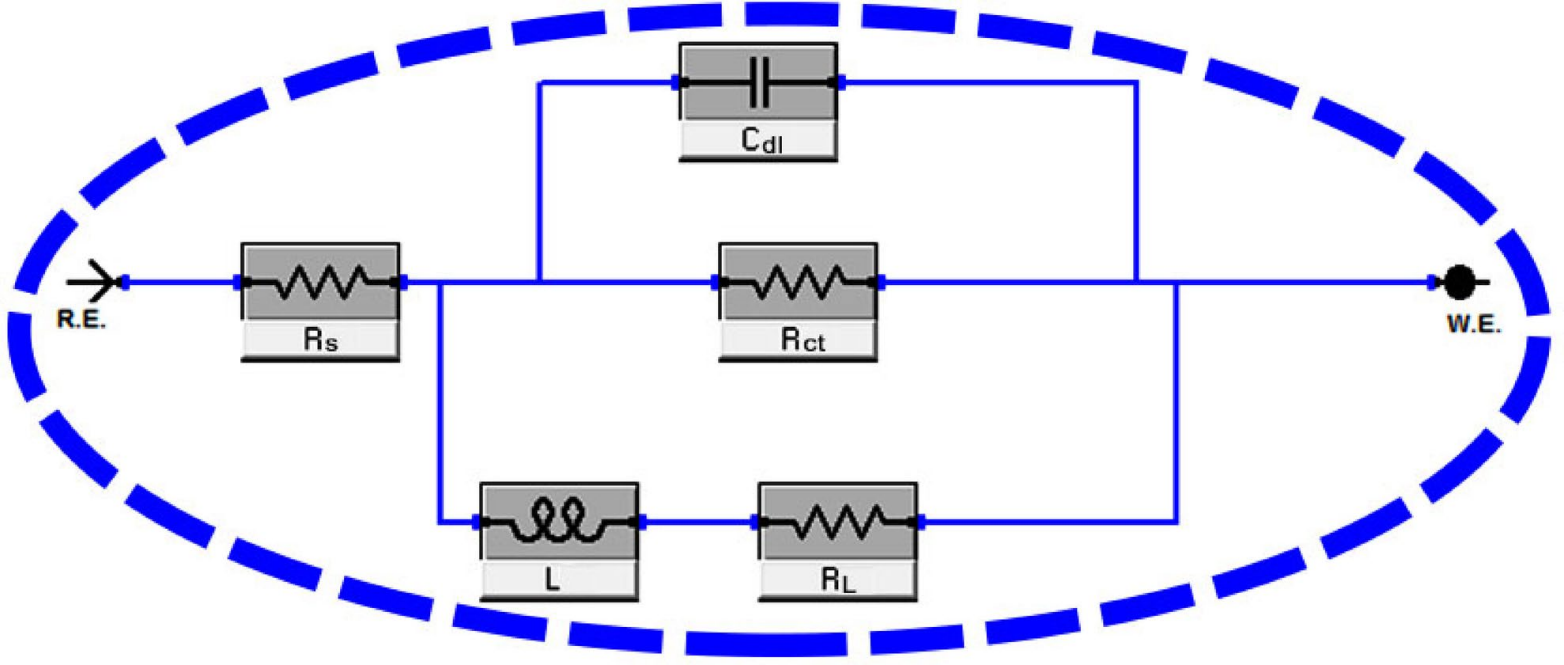
Electrical circuit employed to fit the EIS data.

Figure 11 displays the circuit equivalent model used to display the obtained impedance data. Included in this demonstration are “the double layer capacitance (C_dl_), the inductance (L), the inductive resistance (R_L_), the solution resistance (R_s_), and the charge-transfer resistance of the interfacial corrosion reaction (R_ct_).” The results information fit this demonstration well. When an inductive circle appears, the resistance of polarization can be computed from the subsequent Eq. (14)^67^:

14 $${R}_{P}=\frac{{R}_{ct}\times {R}_{L}}{{R}_{ct}+{R}_{L}}$$

The Bode plots show that higher total impedance (Z) and a shift in phase angle towards larger values correspond with rising CAE concentrations. The adsorption of CAE onto the aluminium surface is responsible for this change. The impedance spectra were examined using an appropriate electrical equivalent circuit, which is shown in Fig. 11. Higher doses of CAE extract cause both R_p_ and R_ct_ levels to rise while C_dl_ values fall, according to an analysis of the EIS data shown in Table 5. This effect is explained by the adsorption of extract molecules on the metal surface, which replaces H_2_O molecules and shows that inhibitor molecules have been adsorbed at the metal/solution contact^68–70^. The inhibition efficiency obtained from EIS tests agrees with the outcome obtained from the PDP and WL techniques.

**Table 5:** EIS data of al in test acidic solution at 25 °C.

[Inh] ppm	*R*_ct_ Ω cm^2^	C_dl_ µFcm^− 2^	*R*_L_ Ω cm^2^	*R*_*p*_, Ω cm^2^	L Hcm^− 2^	θ	%IE
0	15	42	4	5	10	–	–
25	49	12	7	8	16	0.693	69.3
50	62	10	9	10	19	0.758	75.8
75	75	9	14	12	22	0.8	80.0
100	138	8	15	14	26	0.891	89.1
125	143	7	16	26	28	0.895	89.5
150	168	6	33	36	31	0.911	91.1

### Surface examination analysis

#### Scanning electron microscopy (SEM)

**Fig. 12:**
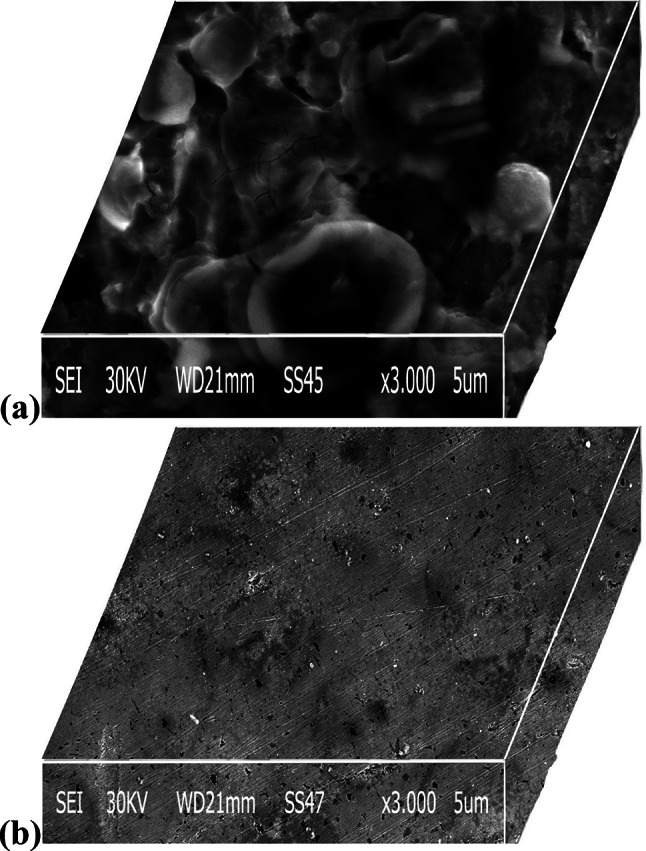
(**a**,**b**) SEM micrographs for Al after exposure to 1 M HCl for one day (**a**) and in the presence of 150 ppm of *CAE* (**b**).

SEM we used to complete and confirm the corrosion inhibition results^71,72^. Optical microscopy images of Al after 24 h of immersion in HCl without and with the compound CAE at an optimum concentration of 150 ppm are shown in Fig. 12. SEM reveals the protective effect of CAE on the Al surface. SEM observations were made on Al samples before and after adding CAE. By comparing the surface finish between the samples that undergo any attack of acid (Fig. 12a) and those treated (Fig. 12b). Figure 12a shows the severe etching caused by the harsh hydrochloric acid environment (1.0 M HCl). However, Fig. 12b demonstrates a significant improvement: the surface treated with CAE exhibits a smoother texture with minimal pits. On the other hand, in the presence of an inhibitor, the surface morphology shows an excellent inhibition compared to Fig. 12a due to the addition of CAE, which forms a protective film against corrosion adsorbed on the surface.

#### Atomic force microscope (AFM)

**Fig. 13:**
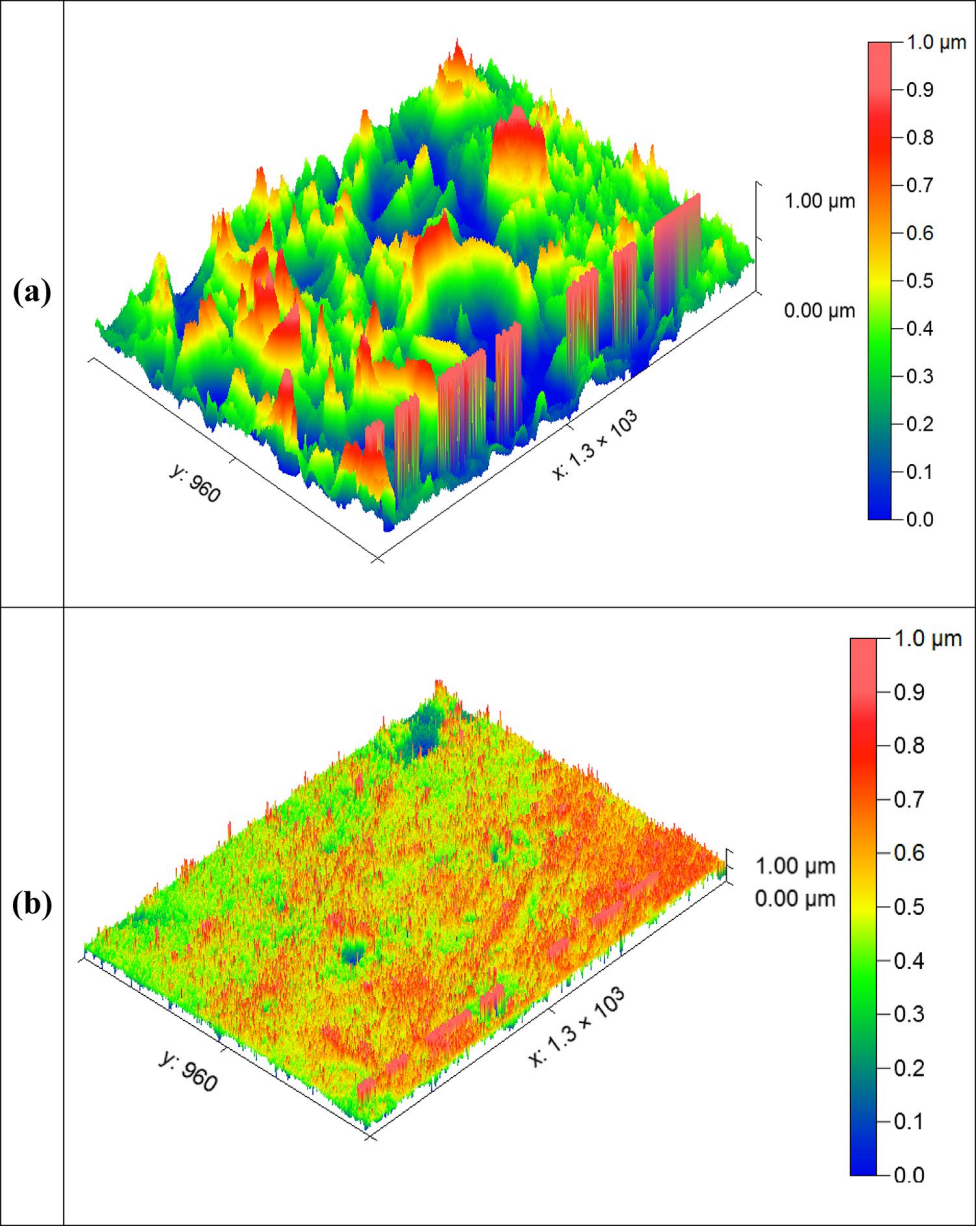
AFM images of Al sample (**a**) in HCl and (**b**) in HCl, including 150 ppm of CAE.

Atomic Force Microscopy (AFM) was employed to gain more precise surface roughness measurements. AFM was used to examine the surface analysis of Al metal immersed in 1 M HCl solution for 24 h at 25 °C with and without CAE inhibitor. The AFM images of the Al surface in Fig. 13a reveal a significant rough surface in a blank solution (without inhibitor) due to the acid solution’s corrosive attack. In contrast, Fig. 13b shows small pits and cavities that indicate that a thin protective layer forms on the metal surfaces when the right amount of inhibitors is added, thereby reducing corrosion. The surface is significantly Average roughness (R_a_) in blank solution (1 M HCl), indicating corrosion 618.3 nm. However, when added 150 ppm of CAE inhibitor, the surface of Al became smoother compared to the uninhibited sample (94.7 nm), indicating that a protective film had formed due to the molecules of CAE extract adsorbed to the Al surface^73^.

### Theoretical calculations

#### Quantum chemical calculation (HOMO-LUMO)

Quantum chemistry analyzes interactions between inhibitor molecular orbitals and Al atomic orbitals. Electronic properties are revealed with the DFT method (E_HOMO_, E_LUMO_, and Dipole moment). A larger E_HOMO_ value indicates a molecule’s ability to donate electrons.

**Fig. 14:**
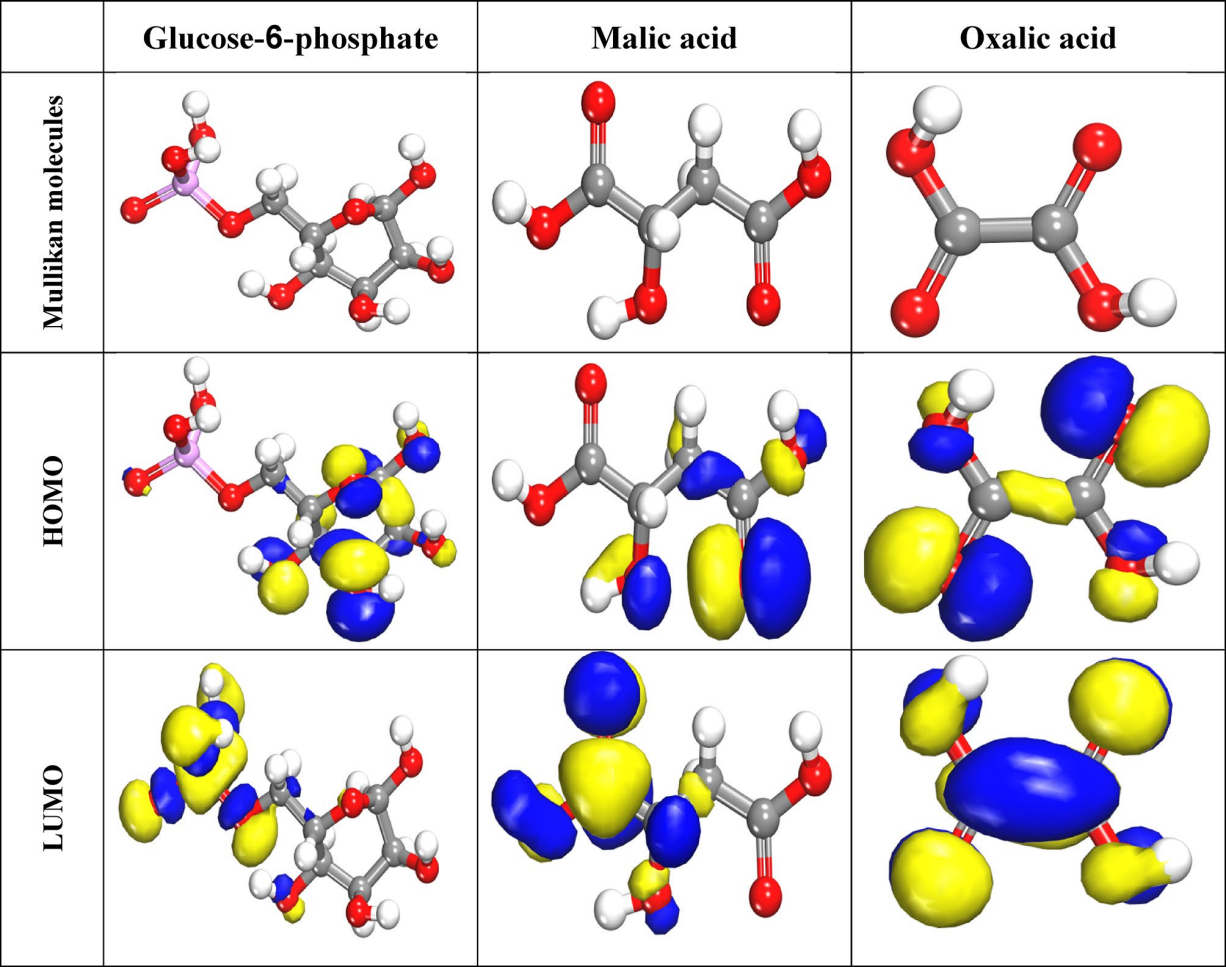
HOMO and LUMO orbitals for three major CAE compounds (Material Studio software (version 7.0) https://www.fullversiondl.com/accelrys-materials-studio-v7-0/).

Figure 14 shows LUMO & HOMO orbitals for the major constituents of CAE extract enclosed Glucose-6-phosphate, Malic acid, and Oxalic acid compounds. E_HOMO_ signifies the capability for electron donation, while E_LUMO_ denotes the capability for electron acceptance. A molecule is more able to donate electrons if its E_HOMO_ is higher and its negative energy value is smaller. The inhibitory potential of a molecule is linked to its dipole moment (*µ*). As µ increases, adsorption also increases. The low energy difference observed and high dipole moment value leads to electrons being transferred from the molecule to the surface. As previous study, decreased global hardness (η) correlates with increased inhibitor reactivity. Molecular stability and reactivity depend on molecules’ absolute hardness (η) and softness (σ)^74^. Table 6 demonstrates E_LUMO_, E_HOMO_, dipole moment (µ), and energy gap (ΔE) for CAE constituents. The investigated CAE component is highly reactive, as indicated by its reduced energy gap (∆E) values, leading to a higher inhibitor efficiency.

Consequently, as Table 6; Fig. 13 demonstrate, CAE components have the highest adsorption tendency on the surface of Al.

15 $${\text{I}}_{\text{p}}\left(\text{i}\text{o}\text{n}\text{i}\text{z}\text{a}\text{t}\text{i}\text{o}\text{n}\:\text{p}\text{o}\text{t}\text{e}\text{n}\text{t}\text{i}\text{a}\text{l} \right)=-{\text{E}}_{\text{H}\text{O}\text{M}\text{O}}$$

16 $$\left(\text{e}\text{l}\text{e}\text{c}\text{t}\text{r}\text{o}\text{n}\:\text{a}\text{f}\text{f}\text{i}\text{n}\text{i}\text{t}\text{y} \right) =-{\text{E}}_{\text{L}\text{U}\text{M}\text{O}}$$

17 $$\chi \: (\text{electronegativity})= \frac{-({E}_{LUMO} + {E}_{HOMO})}{2}$$

18 $$\upmu \:(\text{potential})= -\chi=\frac{\left({E}_{LUMO} + {E}_{HOMO}\right)}{2}$$

19 $$\upeta \: (\text{hardness}) = \frac{({E}_{LUMO}- {E}_{HOMO})}{2}$$

**Table 6:** Quantum analysis of the main ingredients in CAE extract.

Parameters (Variable)	Glucose-6-phosphate	Malic acid	Oxalic acid
E_HOMO_ (eV)	− 6.132	− 6.0292	− 7.665
E_LUMO_ (eV)	− 4.505	− 3.702	− 5.722
∆E, (eV) (E_L_–E_H_)	1.63	2.33	1.94
E_A_ (eV)	6.13	6.03	7.67
I_p_ (eV)	4.51	3.70	5.72
(eV)χ (electronegativity)	5.32	4.87	6.69
µ	− 5.32	− 4.87	− 6.69
η, eV	0.81	1.16	0.97
σ, eV	1.23	0.86	1.03
ω, eV	17.39	10.17	23.06
Δ*E*_back-donation_	− 0.20	0.29	0.24
Dipole moment (debye)	12.463	7.388	4.747
Molecular surface area, Å2	248.712	161.61	117.65

The definition of softness is the global hardness inverse, which is as follows:

20 $$\upsigma\: (\text{softness}) = 1/\eta$$

21 $${\upomega }\left(\text{e}\text{l}\text{e}\text{c}\text{t}\text{r}\text{o}\text{p}\text{h}\text{i}\text{l}\text{i}\text{c}\text{i}\text{t}\text{y}\:\text{i}\text{n}\text{d}\text{e}\text{x}\right) = \frac{{{\upmu }}^{2}}{2{\upeta } }$$

22 $${\varDelta \text{E}}_{back\:donation} =- \frac{{\upeta}}{4}$$

**Fig. 15:**
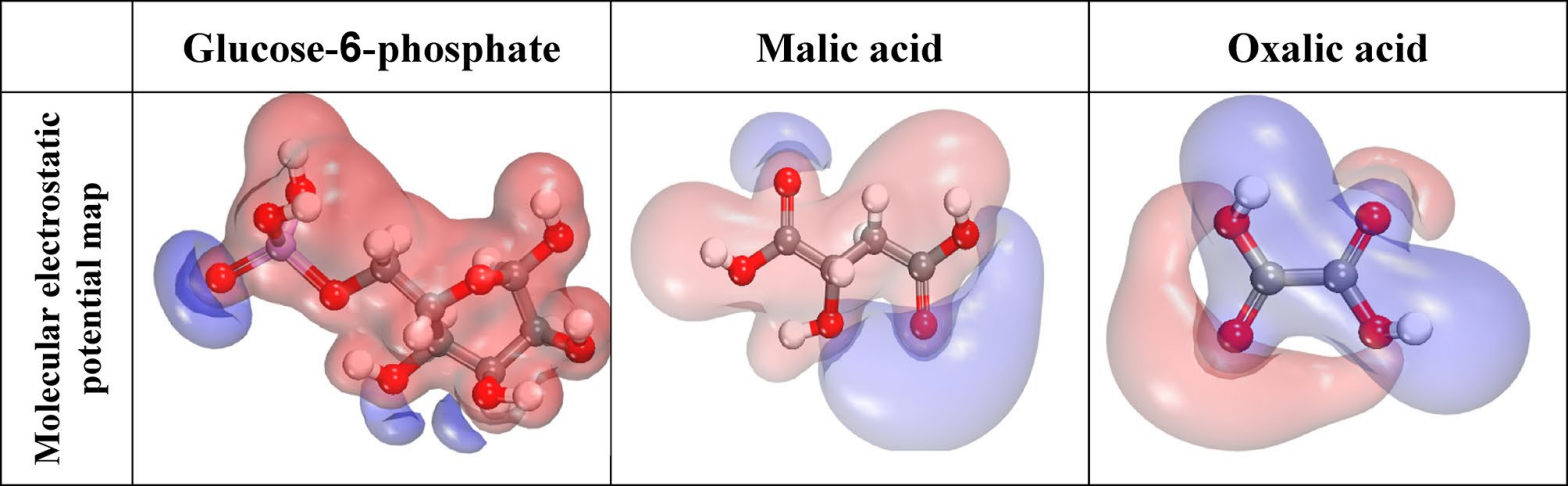
MEP map of the major constituents of CAE extract compounds under investigation (Material Studio software (version 7.0) https://www.fullversiondl.com/accelrys-materials-studio-v7-0/).

The molecular electrostatic potential (MEP) is linked to electron density and is a valuable tool for identifying the locations of electrophilic and nucleophilic processes. The MEP is linked to electron density and is a valuable tool for identifying electrophilic and nucleophilic reaction sites. We used the optimized geometry of major constituents of CAE extract enclosed Glucose-6-phosphate, Malic acid, and Oxalic acid compounds. The MEP map’s light blue and blue colors indicate the nucleophilic active region. The electrophilic active region is shown in red and yellow^75^. The yellow and red lines in the MEP contours indicate the positively and negatively charged areas, respectively. Figure 15 indicates that regions of elevated electron density are located between the heteroatoms and the conjugated double bonds.

#### Monte Carlo (MC) simulation

**Fig. 16:**
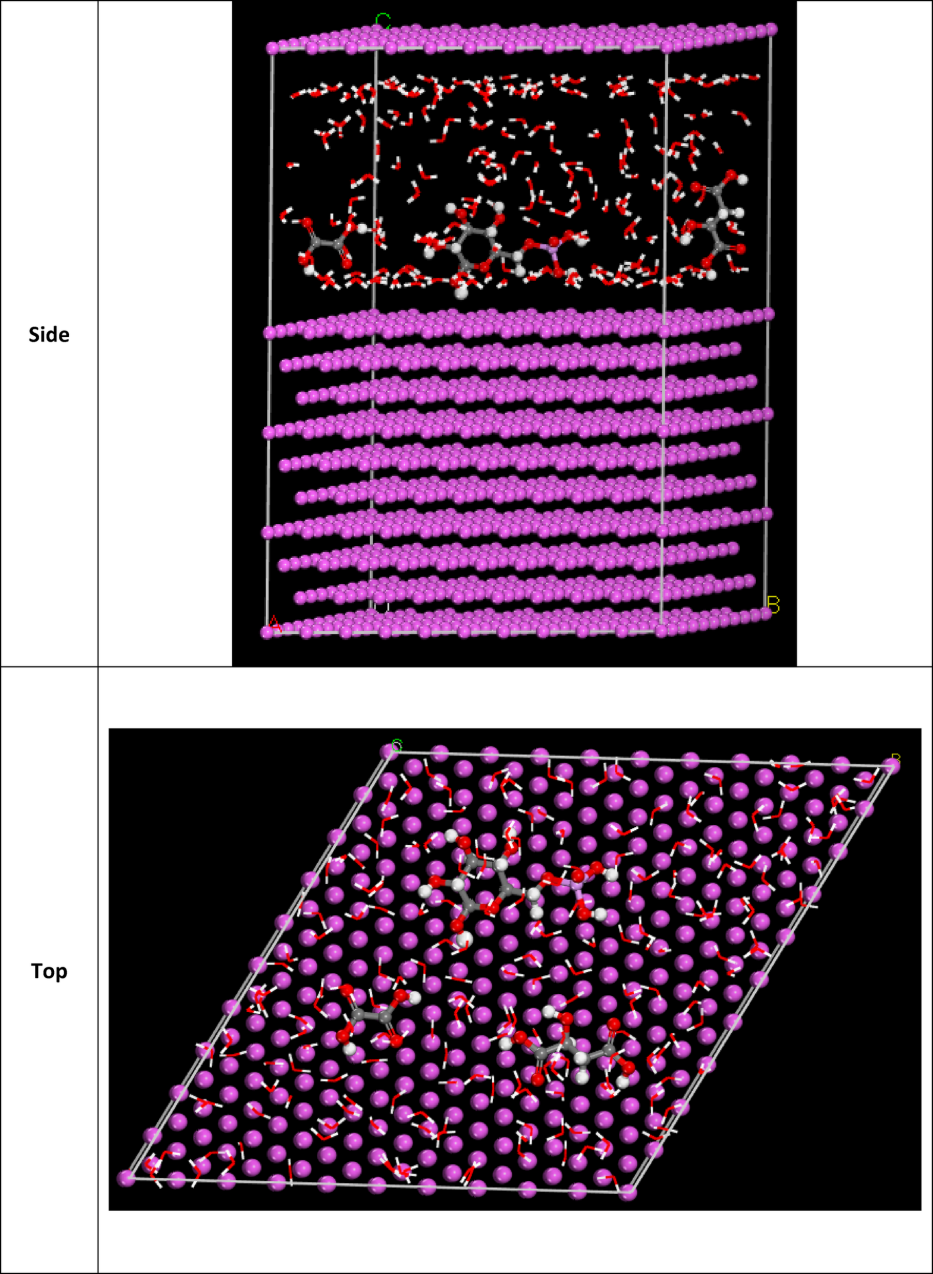
The adsorption locator module produced the most suitable configuration for the adsorption of three major molecules (CAE) on Al (Final Equilibrium configurations) on the Al (111) (Material Studio software (version 7.0) https://www.fullversiondl.com/accelrys-materials-studio-v7-0/).

**Fig. 17:**
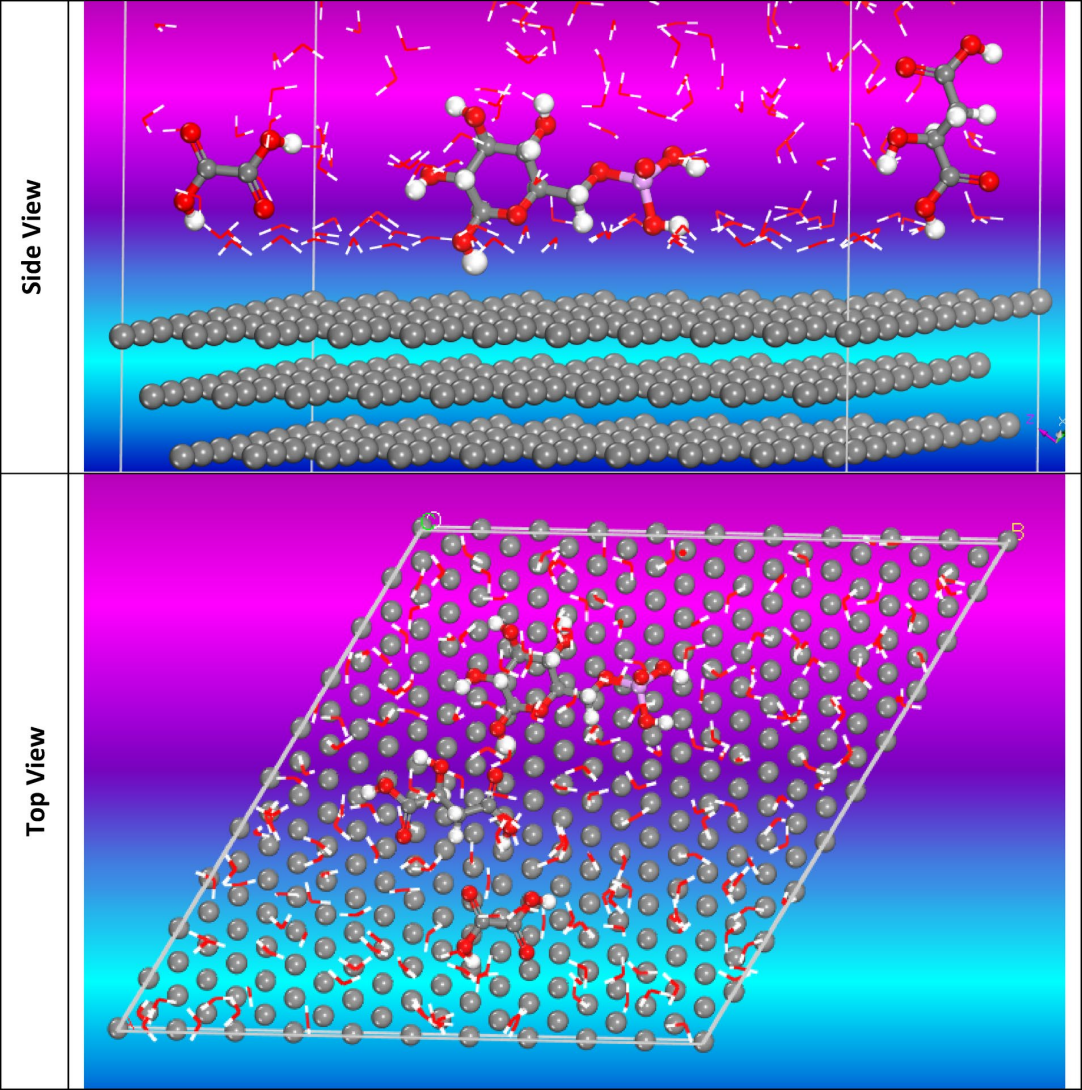
Top and side views show the optimal arrangement for CAE adsorption on the surface of Al (111).

The side (a) and top (b) views of the low energy configuration for adsorbing the major constituents of CAE extract enclosed Glucose-6-phosphate, Malic acid, and Oxalic acid compounds on the Al (111) interface obtained using simulations are presented in Figs. 16 and 17. Our compound is preferentially oriented parallel to the Al (1 1 1) surface, which increases the surface coverage. The adsorption mechanism is due to the π-electrons of the aromatic ring, and the free electrons in the molecule occupy the empty orbitals of the Al and form a protective film on the surface of the metal. The reactivity of Al (1 1 1) seems to be at the beginning of the dipole-dipole interaction, which gives a very stable parallel adsorption structure and a mode of apparent chemisorption + physisorption. The adsorption energy, expressed in kcal mol^−1^, is the energy released or used during the adsorption of the relaxed adsorbate component onto the substrate surface^76^. It finds adsorption centers with the lowest energy on Al surfaces. Surface adsorption leads to a protective thin organic layer that decreases the corrosion rate of the metal. The tabulated display higher adsorption energies (− 4054.908, − 4060.12, − 3550.298 kcal mol^−1^ for Glucose-6-phosphate, Malic acid, and Oxalic acid compounds, respectively. The outputs show that the three compounds are highly efficient adsorptive inhibitors. The tabulated simulation findings in Table 7 confirm that the tested CAE extract adsorbs at a high rate on the Al surface.

**Table 7:** MC parameters obtain form adsorption of CAE on Al (111) (Material studio software (version 7.0) https://www.fullversiondl.com/accelrys-materials-studio-v7-0/.

Structures	Total energy	Adsorption energy, kcal mol^−1^	Rigid adsorption energy, kcal mol^−1^	Deformation energy, kcal mol^−1^	Compound dE_ads_/dN, kcal mol^−1^	H_2_O dE_ads_/dNi, kcal mol^−1^
Al (1 1 0) Glucose-6-phosphate/H_2_O	− 496.312	− 4054.908	− 591.579	− 3469.1	− 207.676	− 17.45
Al (1 1 0) Malic acid/H_2_O	− 559.061	− 4060.12	− 568.607	− 3491.0	− 218.1	− 17.60
Al (1 1 0) Oxalic acid/H_2_O	8.2980	− 3550.298	− 87.251	− 3463.047	− 179.5	− 16.49

### Inhibition mechanism

The two main forms of inhibitor adsorption are chemical and physical adsorption, which is achieved through the creation of coordination bonds, inhibitor molecules, and metal surface share or transfer charges during chemical adsorption. Conversely, physical adsorption depends on the metal’s electrically charged surface and charged species in the solution. Aluminium has low-energy, unoccupied electron orbitals, whereas inhibitor compounds usually have heteroatoms with π electrons and lone pairs of electrons essential for inhibition. Nevertheless, this electron transfer generates an additional negative charge on the metal surface.

**Fig. 18:**
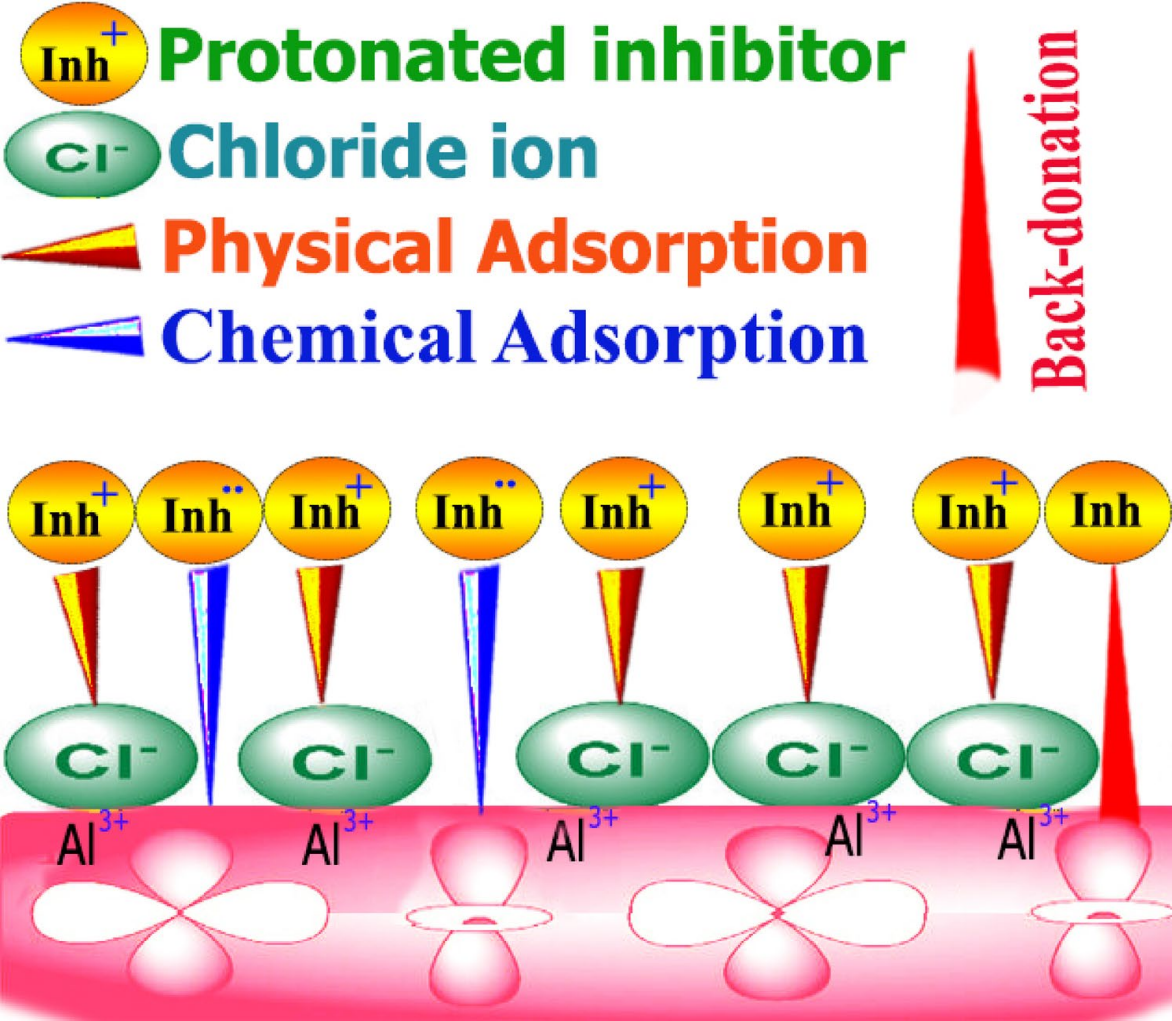
Mechanism of inhibition of CAE on Al surface.

Additionally, this will cause a new transfer (back-donation) to the anti-bonding molecular orbitals of the inhibitor molecules. Figure 18 illustrates the adsorption mechanism of specific CAE extract in HCl on the Al surface under acidic conditions (physical adsorption, chemical adsorption and back donation). Due to the aggressive acidic environment, the Al surface oxidized rapidly and became positively charged. This allowed the attraction of negatively charged chloride anions, resulting in a negative metallic surface. The CAE extract in one molar hydrochloric acid exists mostly as cations (protonated forms). The negatively charged metal surface attracts the protonated inhibitor molecules, causing them to adhere through electrostatic forces^77,78^. Thus, adsorption combined with mainly physical electrostatic interaction is principally responsible for the corrosion inhibition of aluminium in 1 M HCl.

## Conclusions

1. The use of CAE as a corrosion inhibitor shows that, in HCl conditions, this inhibitor is effective against the corrosion of Al.
2. CAE shows significant inhibitory properties against corrosion of Al in one molar HCl solution. The inhibitory effectiveness increases with increasing inhibitor concentration.
3. The inhibition action of the inhibitors suggests that the inhibitor function through adsorption was consistent with the Langmuir Adsorption Isotherm.
4. The values of Δ*G*_ads_^0^ were negative, and this confirmed the spontaneity of the adsorption process.
5. Analysis of PDP data suggested that CAE acts as a mixed-type inhibitor, affecting both anodic and cathodic reactions.
6. Through a comprehensive examination of experimental data along with SEM and AFM analysis, the inhibition mechanism of Al in the aggressive solution was elucidated, providing valuable insights into the inhibitory properties of CAE plant extract on Al corrosion in acidic environments.
7. Theoretical calculations align well with experimental methods, demonstrating the significance of the molecular structure of CAE molecules in inhibiting the corrosive process.

## Data Availability

“Data is provided within the manuscript”.
